# Radiological features of brain hemorrhage through automated segmentation from computed tomography in stroke and traumatic brain injury

**DOI:** 10.3389/fneur.2023.1244672

**Published:** 2023-09-28

**Authors:** Bradley J. MacIntosh, Qinghui Liu, Till Schellhorn, Mona K. Beyer, Inge Rasmus Groote, Pål C. Morberg, Joshua M. Poulin, Maiken N. Selseth, Ragnhild C. Bakke, Aina Naqvi, Amir Hillal, Teresa Ullberg, Johan Wassélius, Ole M. Rønning, Per Selnes, Espen S. Kristoffersen, Kyrre Eeg Emblem, Karoline Skogen, Else C. Sandset, Atle Bjørnerud

**Affiliations:** ^1^Computational Radiology & Artificial Intelligence Unit, Department of Physics and Computational Radiology, Clinic for Radiology and Nuclear Medicine, Oslo University Hospital, Oslo, Norway; ^2^Department of Medical Biophysics, Faculty of Medicine, University of Toronto, Toronto, ON, Canada; ^3^Hurvitz Brain Sciences, Sandra Black Centre for Brain Resilience & Recovery, Physical Sciences Platform, Sunnybrook Research Institute, Toronto, Oslo, Norway; ^4^Division of Radiology and Nuclear Medicine, Oslo University Hospital, Oslo, Norway; ^5^Department of Radiology, Vestfold Hospital Trust, Tønsberg, Norway; ^6^Department of Radiology and Department of Surgery, Vestfold Hospital Trust, Tønsberg, Norway; ^7^Department of Neurology, Akershus University Hospital, Lørenskog, Norway; ^8^Department of Diagnostic Imaging, Akershus University Hospital, Lørenskog, Norway; ^9^Department of Diagnostic Radiology, Neuroradiology, Skåne University Hospital, Lund, Sweden; ^10^Department of Medical Imaging and Physiology, Skåne University Hospital, Lund, Sweden; ^11^Department of Clinical Sciences, Faculty of Medicine, Lund University, Lund, Sweden; ^12^Institute of Clinical Medicine, University of Oslo, Oslo, Norway; ^13^Department of General Practice, Institute of Health and Society, Faculty of Medicine, University of Oslo, Oslo, Norway; ^14^Department of Physics and Computational Radiology, Clinic for Radiology and Nuclear Medicine, Oslo University Hospital, Oslo, Norway; ^15^Department of Neurology, Oslo University Hospital, Oslo, Norway; ^16^Department of Physics, University of Oslo, Oslo, Norway

**Keywords:** computed tomography, intracerebral hemorrhage, stroke, traumatic brain injury, segmentation, deep learning

## Abstract

**Introduction:**

Radiological assessment is necessary to diagnose spontaneous intracerebral hemorrhage (ICH) and traumatic brain injury intracranial hemorrhage (TBI-bleed). Artificial intelligence (AI) deep learning tools provide a means for decision support. This study evaluates the hemorrhage segmentations produced from three-dimensional deep learning AI model that was developed using non-contrast computed tomography (CT) imaging data external to the current study.

**Methods:**

Non-contrast CT imaging data from 1263 patients were accessed across seven data sources (referred to as sites) in Norway and Sweden. Patients were included based on ICH, TBI-bleed, or mild TBI diagnosis. Initial non-contrast CT images were available for all participants. Hemorrhage location frequency maps were generated. The number of estimated haematoma clusters was correlated with the total haematoma volume. Ground truth expert annotations were available for one ICH site; hence, a comparison was made with the estimated haematoma volumes. Segmentation volume estimates were used in a receiver operator characteristics (ROC) analysis for all samples (i.e., bleed detected) and then specifically for one site with few TBI-bleed cases.

**Results:**

The hemorrhage frequency maps showed spatial patterns of estimated lesions consistent with ICH or TBI-bleed presentations. There was a positive correlation between the estimated number of clusters and total haematoma volume for each site (correlation range: 0.45–0.74; each *p*-value < 0.01) and evidence of ICH between-site differences. Relative to hand-drawn annotations for one ICH site, the VIOLA-AI segmentation mask achieved a median Dice Similarity Coefficient of 0.82 (interquartile range: 0.78 and 0.83), resulting in a small overestimate in the haematoma volume by a median of 0.47 mL (interquartile range: 0.04 and 1.75 mL). The bleed detection ROC analysis for the whole sample gave a high area-under-the-curve (AUC) of 0.92 (with sensitivity and specificity of 83.28% and 95.41%); however, when considering only the mild head injury site, the TBI-bleed detection gave an AUC of 0.70.

**Discussion:**

An open-source segmentation tool was used to visualize hemorrhage locations across multiple data sources and revealed quantitative hemorrhage site differences. The automated total hemorrhage volume estimate correlated with a per-participant hemorrhage cluster count. ROC results were moderate-to-high. The VIOLA-AI tool had promising results and might be useful for various types of intracranial hemorrhage.

## 1. Introduction

Spontaneous intracerebral hemorrhage stroke (ICH) and traumatic brain injury hemorrhage (TBI-bleed) are examples of acute brain conditions where rapid imaging is needed for a diagnosis. Non-contrast-enhanced computed tomography (CT) remains the gold standard imaging modality to detect ICH and TBI-bleed. ICH appears to rise with time and accounts for 9 to 26% of acute stroke (1, 2). TBI-bleed contributes a high burden due to the sheer number of head injuries worldwide (3–5).

Radiological features of intracranial hemorrhage provide information that can help with ICH and TBI-bleed diagnosis/prognosis. The total haematoma volume is used in the ICH Score, which is a grading system used clinically (6). The ABC/2 method is a well-established approach to estimate total haematoma volume; the expert human measures the maximum length and width of the haematoma in the axial plane, determines the number of slices where the haematoma is visible, and divides the product of these values by two (7). The ABC/2 method and modified or simplified versions provide good agreement with contoured hand annotation ground truth (8–10). Once a haematoma is segmented, it can be analyzed for its location, shape, number of distinct locations (cluster count), texture, and radiomic features (11–14).

Automated hemorrhage segmentation is desirable for research purposes, and ideally, such a method should be agnostic to the CT imaging protocol. Machine learning and deep learning artificial intelligence (AI) have had a decisive role in this regard, and a recent review discusses commercial solutions (15). Muschelli et al. (16) achieved accurate segmentation results based on a random forest machine learning approach relative to ground truth contoured annotation. Another approach by Chilamkurthy et al. (17) demonstrated how an AI system could provide decision support by detecting hemorrhage-positive cases with an area-under-the-curve (AUC) classification that exceeded 0.90. Automated methods can facilitate radiological discovery, particularly for large sample-sized research cohorts or secondary clinical trial CT imaging data analysis. Furthermore, open-source software can enable the further development of the method.

The current study investigates the segmentation results when a previously trained deep learning neural network called “Voxels Intersecting along Orthogonal-Levels of Attention” (VIOLA-AI) is used on de-novo ICH and TBI-bleed cases (18). There are four aims. First, we created hemorrhage frequency maps for each data sources/sites. Second, we test whether the number of distinct contiguous haematoma clusters that are detected will correlate with the total haematoma volume. Third, we conducted between-site comparisons for the ICH sites/data sources. Fourth, we evaluated classification metrics based on the hemorrhage segmentation through a receiver operator characteristic (ROC) analysis of the entire sample and secondarily the consecutive cases of patients presenting with mild TBI.

## 2. Methods

### 2.1. Participants

The study recruited individuals with a confirmed intracranial hemorrhage (ICH or TBI-bleed) or a suspected brain injury (with or without concomitant bleeding). For inclusion, participants had to be over 16 years old at the time of the assessment and have a non-contrast brain CT scan available. Data were grouped from seven different sources, either sites or research projects (herein referred to as “sites”). All sites provided original non-contrast CT head scan images in either DICOM or NIfTI format. Any data of low quality, unusable due to artifact after visual inspection, or consisted of secondary capture DICOM files were excluded from the analysis. The respective research ethics committees approved data collection at each site. One public data repository was also considered as a site. This study benefitted from an external dataset of 100 diagnosed intracranial hemorrhage cases used in the deep learning model training (details below).

### 2.2. Sites

Table 1 provides summary details on the different sites. Additional details are provided herein (co-author names are provided as initials).

**Table 1:** Study characteristics are provided.

	**ICH**				**Mixed**	**TBI**	
**Group**	**Akershus**	**Skåne**	**NorCoast**	**Ullevål**	**CQ500**	**Ullevål**	**Oslo Emergency**
N	65	223	57	53	40	130	695
Mean haematoma volume mL and range	26.44; 0.02 to 210.32	37.28; 0.00 to 239.80	20.11; 0.00 to 134.91	47.95; 0.24 to 241.55	37.41; 0.00 to 151.90	24.79; 0.00 to 197.57	0.37; 0.00 to 36.20
Mean number of clusters (range)	2.66; 1.00 to 21.00	3.68; 0.00 to 23.00	2.18; 0.00 to 11.00	3.79; 1.00 to 17.00	9.38; 0.00 to 31.00	4.39; 0.00 to 29.00	0.63; 0.00 to 13.00

The first four columns were ICH sites/data sources. CQ500 data were ICH and TBI-bleed cases. The last two columns were TBI sites. Mean estimated total haematoma volume and number of distinct locations (i.e., clusters) are provided. See Supplementary Table 1 for imaging details.

***Akershus:*** 65 ICH cases were accessed from the Akershus University Hospital as part of an on-going stroke project that is led by ESK, OMR, PS. Ground truth segmentations were available for this site.

***Skåne:*** 223 ICH cases were selected from an existing stroke database at the Skåne University Hospital in Lund/Malmö, Sweden (9) and cases were overseen/reviewed by JW and TU.

***NorCoast:*** 57 ICH cases were accessed from a multi-site stroke research study called NorCoast. One senior neuroradiologist (TS) was involved in curating these data and reviewing ICH diagnosis. These ICH patients were accessed clinically at St. Olav's Hospital in Trondheim, Norway, or Vestre Viken Hospitals in Bærum, Norway.

***Ullevål stroke:*** 53 ICH were accessed from the Ullevål site of Oslo University Hospital and the ICH diagnosis was reviewed by KS and ECS.

***CQ500:*** 40 CT cases were randomly selected from the open-access CQ500 database (19). These cases were previously reviewed by three clinicians linked to CQ500. Based on the tabular notes (filename: cq500_reads.csv), at least one radiologist scored ICH in all 40 of these cases where 31 of which were deemed intraparenchymal, and intraventricular hemorrhage was noted in as many as 23 cases.

***Ullevål TBI:*** 130 confirmed TBI bleed cases were accessed from Oslo University Hospital Ullevål and cases were overseen/reviewed by KS.

***Oslo Emergency Unit TBI:*** 695 cases of suspected TBI were accessed from the Oslo Accident and Emergency Outpatient Clinic, Oslo University Hospital, a rapid assessment unit equipped with a CT scanner. This group consisted of consecutive patients that presented with suspected TBI between (between 01.01.2016 and 14.04.2016). Cases were overseen/reviewed by KS and inspected for imaging artifacts by BJM. The anticipated rate of TBI-bleed cases for these “walk in” patients was expected to be low frequency.

### 2.3. Image data and analysis

CT images were collected according to the clinical acute head protocols at the respective sites. The acquisition details tended to vary in spatial resolution and head coverage. The VIOLA-AI model was previously tested and developed as part of the INSTANCE 2022 intracranial hemorrhage segmentation challenge (18). The CT external training data consisted of 100 cases of intraparenchymal and intraventricular hemorrhage cases, including subdural, epidural, and subarachnoid hemorrhages. Ground truth segmentations were provided by the INSTANCE 2022 organizers and inspected visually by neuroradiologists from the current study (KS, TS). Images were reconstructed with voxel dimensions of 0.45 mm x 0.45 mm x 5.0 mm and the following ranges: x-resolution: 0.37 to 0.60 mm; y-resolution: 0.37 to 0.60 mm; z-resolution: 4.40 to 7.07 mm.

Ten models based on three-dimensional neural networks were trained, with five-fold validation and a test set to yield the Viola-AI tool ensemble. The first five models were implementations of the no-new U-Net (nnU-Net, monai.io); these models were solely trained with hyperparameter tuning without architectural changes. Five more models were trained after incorporating attention modules during the decoding branch of the U-Net. This architecture is called “Voxel Intersecting along Orthogonal Levels Attention U-Net” (VIOLA-U-Net). The architecture was specifically designed to make segmentations for hemorrhage-positive cases. Model training was performed on the Digital Research Alliance of Canada's high-performance cluster. We used four metrics to appraise the performance of the VIOLA-AI model: dice similarity coefficient, Hausdorff distance, normalized surface dice, and relative absolute volume difference. For the current study, no new training was performed. We refer to the ensemble of models as VIOLA-AI tool.

Original CT data were accessed and stored on local secure servers. The VIOLA-AI tool was run as a docker (Docker, Inc., Palo Alto, CA), with each site considered as a batch and invoking a graphics processing unit to calculate each segmentation mask as an inference. Each segmentation was saved as a binary mask in a prediction folder.

Since these data had high spatial resolution images, a group template CT image was created from the Akershus site using the Advanced Normalization Toolkit (antsRegistration). The CT group average template was oriented into MNI152 standard space to facilitate mask overlays. Original CT images were linearly registered to the group template using an affine registration tool called FLIRT with 12 degrees of freedom as part of the FMRIB Software Library. The transformation matrix (original CT to group average standard space) was applied to the corresponding binary mask segmentations. An average segmentation mask was generated per site for an estimated frequency map. This enabled visualization of the average locations of suspected hemorrhages.

Two radiological metrics were calculated per patient: the total estimated haematoma volume and the number of distinct haematoma clusters. For the former, the number of voxels in the segmentation mask was multiplied by the voxel volume for the given acquisition to yield volume units (mL). For the latter, we note that an ICH and TBI-bleed can produce distinct groups of voxels that make up individual haematoma clusters, which we determined using a cluster command provided by in FMRIB Software Library.

### 2.4. Statistics

We calculated the centroid location based on the average number of clusters and total haematoma volume to summarize the VIOLA-AI results per site. Correlations were performed per site to test whether the total estimated haematoma volume was associated with the number of distinct haematoma clusters. We conducted a non-parametric Kruskal-Wallis Rank Sum for the four ICH sites to test for a site effect based on median total haematoma volume estimates. The Kruskal-Wallis test for a site effect was also performed for the median cluster number. A *p*-value of 0.05 was considered significant. For one ICH site, we quantified the difference in haematoma volume (ground truth—estimate) vs. the average haematoma volume of the estimate and ground truth.

Receiver operator characteristic (ROC) analysis was performed for: (a) the entire sample and (b) the consecutive suspected TBI cases from the Emergency Oslo Unit. The purpose was to evaluate the classification performance metrics between hemorrhage-positive vs. hemorrhage-negative individuals, e.g., for the Emergency Oslo Unit, this classification amounted to TBI-related hemorrhage: yes/no. The statistics were performed using R (R version 3.6.2, R Core Team) “pROC” library to produce the following estimates: area-under-the-curve (AUC), sensitivity, specificity, positive predictive value (PPV), and negative predictive value (NPV).

## 3. Results

**Figure 1:**
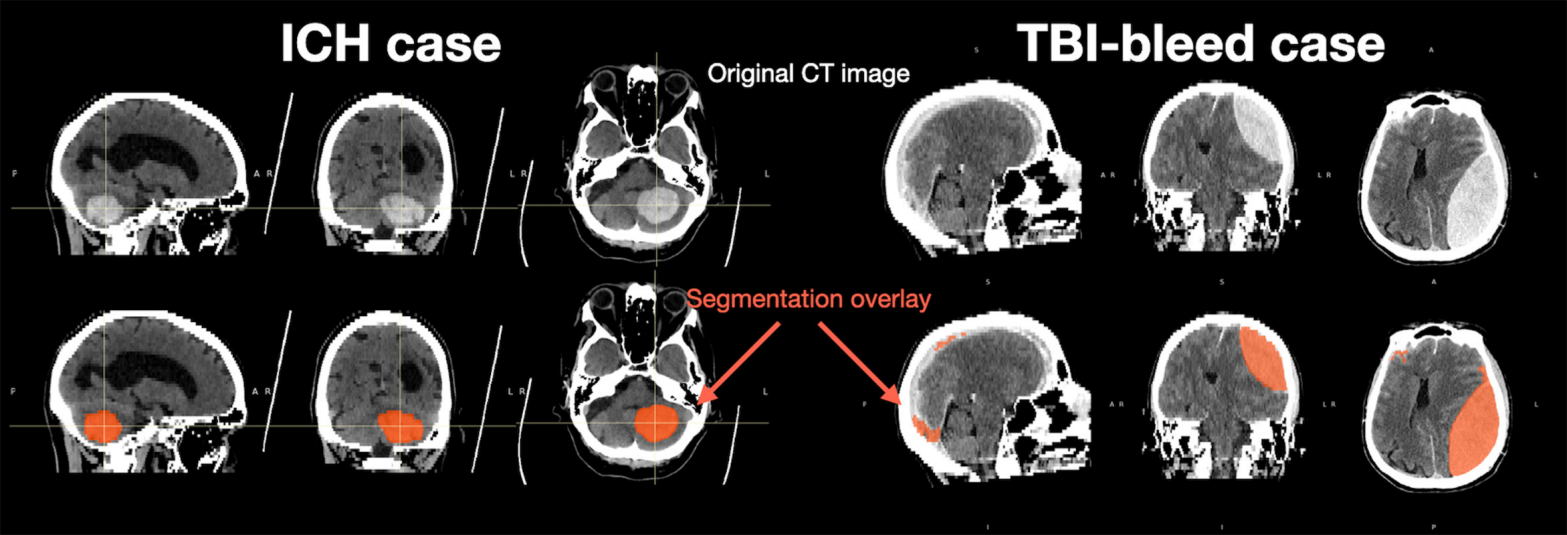
Representative data from ICH **(left)** and TBI-bleed **(right)** cases. The original CT images are shown in the top row and the bottom row shows the Viola-AI-based segmentation in red.

The VIOLA-AI tool produced segmentation estimates in all cases. A single segmentation inference took 16 sec using a graphics processing unit (standard deviation: 2.7 sec). Figure 1 shows the segmentations for one ICH and one TBI case. Table 1 shows haematoma summary information (Table 2).

**Table 2:** Summary of performance metrics for the VIOLA-AI tool that was trained on 100 diagnosed ICH and TBI-bleed cases and evaluated using five-fold cross validation.

**Model**	**Dice similarity coefficient**	**Hausdorff distance**	**Normalized surface dice**	**Relative absolute volume difference**
VIOLA-AI ensemble	0.7953 ± 0.172	21.557 ± 25.021	0.5681 ± 0.125	0.1980 ± 0.180

Performance metrics were calculated from 20 cases held out during each of the five folds. The Dice Similarity Coefficient, for instance, reached an average level that indicates good segmentation agreement between the estimated and ground truth.

**Figure 2:**
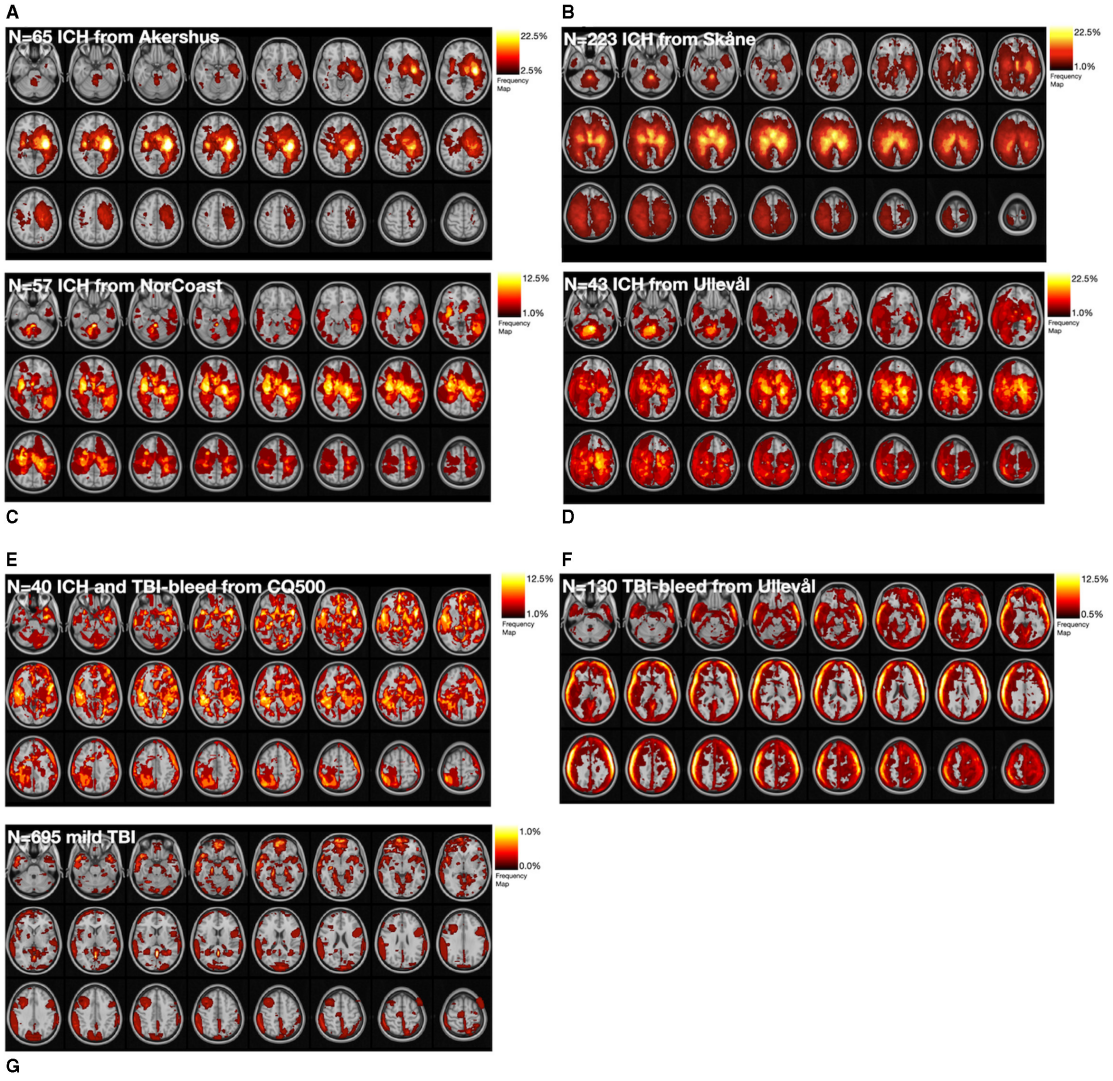
Binary lesion segmentation masks were overlaid on a reference coordinate space to visualize the average location of the hemorrhage on a per site basis. **(A–D)** show the results for the four ICH sites. **(E)** shows the results from the open access CQ500 ICH and TBI-bleed cases. **(F)** is from the confirmed TBI-bleed site, while **(G)** is the consecutive cases of suspected TBI. Probability values are provided using a heat map colour panel in the top right corner for each panel.

Figure 2 shows the estimated hemorrhage segmentation masks (per site or data source) in standard space and it is possible to visually discern site differences. The ICH sites tended to show hemorrhage segmentations in deeper brain structures, while TBI sites tended to show haematoma locations along the surface of the brain. The low number of hemorrhage cases for the Emergency Oslo Unit site is reflected in the color map with the lowest frequency for the group.

**Figure 3:**
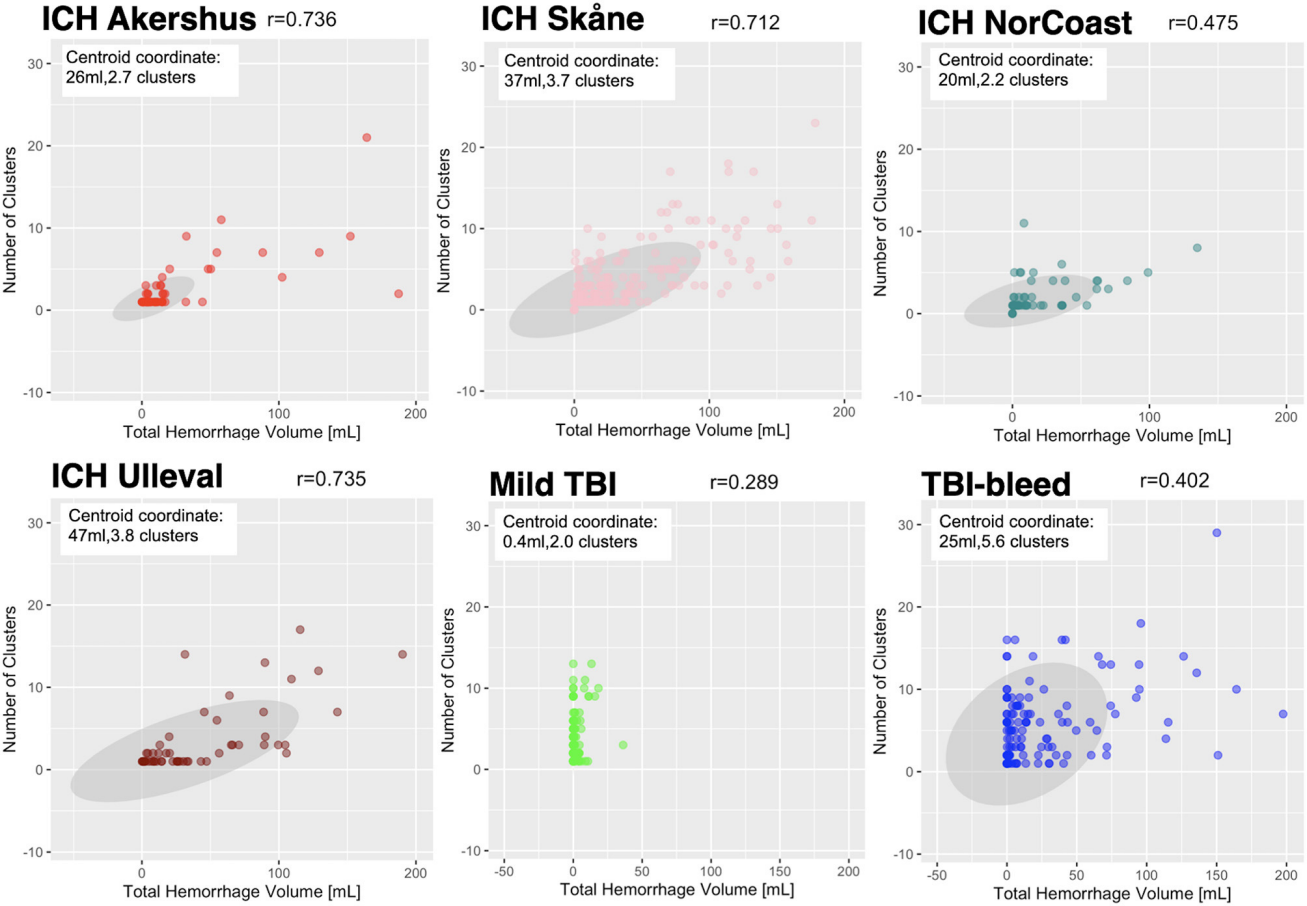
The number of detected clusters is plotted against the total hemorrhage volume per site. Information on the site name and patient group are provided in each panel title. Each data point corresponds to an individual patient. Each panel shows a greyscale ellipse that is the 95% confidence interval for the site and the centroid coordinate is provided. Bivariate correlation coefficients were calculated per site and a range of correlations were observed (lowest r = 0.289, highest r = 0.736); all correlations were statistically significant (*P* < 0.01).

Figure 3 shows scatterplots for four ICH sites and two TBI sites. All groups showed a significant relationship between the number of clusters and total haematoma volume; the correlation coefficients ranged from 0.289 to 0.736 (*p*-values for each site were < 0.01). For the four ICH sites, the Kruskal-Wallis test (chi-squared = 19.294 and degrees of freedom = 3) revealed a significant site effect with p-value = 0.000238; i.e. the Ullevål ICH site had the highest median total haematoma volume relative to three other ICH sites. The Kruskal-Wallis test (chi-squared = 12.636, degrees of freedom = 3) also showed a site effect for the number of haematoma clusters with a *p*-value = 0.00549.

For one ICH site, the agreement between estimated and ground truth masks was high with a median Dice Similarity Coefficient value of 0.82, and the interquartile range was 0.777 to 0.863. The median difference in the haematoma volume (ground truth—estimated) was −0.47 mL (interquartile range of −1.75 and −0.04 mL, and maximum overestimate and maximum underestimate of −93.19 and 3.09 mL).

The AUC for the entire sample was 0.92 based on a haematoma minimum volume threshold of 0.69 mL. The sensitivity and specificity were 83.28 and 95.41%, respectively. The PPV and NPV were 95.73 and 82.21%, respectively. Considering only the Oslo Emergency Unit, we removed cases with imaging motion or metallic artifacts (*n* = 25) and duplicates (*n* = 109), resulting in *n* = 564 cases suitable for the secondary ROC. For this site, the AUC was 0.69, which was based on classifying 33 cases (out of 37 diagnosed) as TBI-bleed while the remaining 531 were hemorrhage negative. The hematoma minimum volume threshold was 0.34 mL, with sensitivity and specificity of 95.92 and 25.00%, respectively, and PPV and NPV that were 91.99 and 40.54%, respectively.

## 4. Discussion

In the current study we used the VIOLA-AI tool to segment hemorrhage location among patients with ICH, TBI-bleed, or suspected TBI-bleed. This study involved multiple data sources as a means of characterizing radiological features of intracranial hemorrhage. The frequency maps revealed spatial patterns for hemorrhage locations that were consistent within disease groups. These maps were suggestive of between-site differences, as reflected in significant between-site differences in the total haematoma volume specifically among the four ICH sites. Each site demonstrated a strong positive association between the number of haematoma clusters and the estimated haematoma volume. Manual annotations were performed at one ICH site and suggestive of a good agreement with ground truth. The VIOLA-AI segmentation tool achieved good classification performance as reflected by the ROC area-under-the curve for the total sample and the site with consecutive suspected TBI.

We note that the estimated ICH locations tended to include lobar, basal ganglia, thalamus, internal and external capsule regions. The spatial patterns observed in the current study tended to align with previous work, although it should be noted there were limited estimates in infratentorial locations (20–22). The most frequent locations for the ICH segmentations (in standard space) did not exceed 25% of the sample at any given site. This finding tends to align with work by others, who collectively reported on supratentorial locations, putamen, and thalamus as being common ICH locations, in addition to the cerebral hemispheres and ventricle locations (9, 23, 24). Future work is needed to characterize the locations of ICH in relation to radiological notes because there is renewed interest in location specific haematoma volume cut-offs in relation to outcomes (14).

The TBI frequency maps' spatial pattern was markedly different compared to ICH. The ring-like pattern near the skull is to be expected, and there were no locations that exceeded 12% of the sample site. Others report there is a propensity for TBI-bleeds to be superficial lesions in frontal, temporal, orbitofrontal and anterior temporal regions (25). Mild TBI tends to be in frontal and temporal regions while others report more diffuse patterns for subdural haematomas (26); these spatial patterns are relatively consistent across studies (27).

The current study included an estimate of the number of distinct hemorrhage clusters, a by-product of the VIOLA-AI segmentation tool. In doing so, we observed robust associations between the number of clusters and the estimated total haematoma volume. ICH is often characterized by a primary parenchymal hemorrhage that can expand into other locations and/or tissue spaces; hence multiple clusters are evident. Up to 43% of ICH will involve intraventricular hemorrhage, which indicates that multiple hemorrhage clusters are expected (24). Indeed, intraventricular hemorrhage is an independent factor associated with mortality and morbidity and for which a radiological scoring system has long since been established (10). Future work is needed to relate the binary segmentation masks in terms of their underlying and potentially multi-focal tissue constituents. More research is needed to connect the radiological-derived features with clinical variables as well as biophysical hemorrhage models that explain spatial expansion patterns (28).

The discrepancy between estimations and ground truth lesion volumes from the ICH Akershus site was small based on the median difference. There was one clear case of a failed segmentation for this site where the discrepancy between the VIOLA-AI estimate and ground truth was very large and constituting an overestimate by the model. Future work is needed to determine the factors contributing to these discrepancies and whether the VIOLA-AI volume estimates can improve.

The ROC analysis for the entire sample showed high performance metrics, while the performance metrics were lower for the suspected TBI site. Several other studies have reported on hemorrhage detection using CT scans (9, 19) and these efforts used considerably larger sample sizes than the external CT cases for the current VIOLA-AI tool. It is intriguing that the VIOLA-AI tool achieved adequate classification performance for all samples, but the scores decreased when considering the low yield cases of consecutive mild TBI cases (29). This suggests that VIOLA-AI is currently better suited for ICH and confirmed TBI-bleeds rather than as a detection tool for mild TBI sites.

The current study has novel elements as well as limitations. The VIOLA-AI tool was trained using a three-dimensional neural network architecture; hence, the entire 3-dimensional ground truth annotation mask was considered during model training, which differs from segmentation tools using a 2-dimensional model (30). The VIOLA-AI tool is an ensemble of ten separately trained deep-learning neural networks, which may lead to more robust/consistent results. No model was re-trained for the data in the current study. This research adds to the growing AI literature of CT-based ICH segmentation (31–33). On-going efforts are needed to improve performance of TBI-bleed, which can be challenging to detect and segment due to their close proximity to the skull giving high attenuation artifacts, or are more subtle when confined to one sulci. An advantage of VIOLA-AI is that original DICOM data were used, and no pre-processing was needed beyond converting the images to NIfTI format. Although the INSTANCE2022 Data Challenge external data were relatively diverse, it was not balanced for all the forms of intracranial hemorrhage. Hence, re-training would be advisable by including more epidural, subdural hematomas, and subarachnoid hemorrhage cases. Another limitation is that only non-contrast CT images were considered; segmentation estimates are likely to fail for images containing signals from exogenous contrast. Another limitation is the data were restricted to the initial CT; repeat imaging was beyond the scope of the current study.

In conclusion, several data sources were accessed and demonstrated that the VIOLA-AI tool was capable of segmenting ICH and TBI-related hemorrhage on non-contrast CT images. We found a consistent relationship between hemorrhage volume and cluster count, and these radiological features significantly differed between the ICH sites. It is important to determine whether inclusion criteria, patient demographics, disease severity, or other health service factors influenced these differences. An example of future work is multi-class hemorrhage segmentation (9, 34, 35) because hemorrhage location is important for prognosis and potential treatment efficacy (36). The hemorrhage frequency maps were overlaid on a standard coordinate space atlas and can help assess lesion locations. Running the VIOLA-AI tool in a batch mode would be conducive to analysis of existing CT images that were collected as part of ICH or TBI clinical trials.

## Data availability statement

The raw data supporting the conclusions of this article will be made available by the authors, without undue reservation.

## Ethics statement

The studies involving humans were approved by Research Ethics Council for Oslo University Hospital. The studies were conducted in accordance with the local legislation and institutional requirements. The participants provided their written informed consent to participate in this study.

## Author contributions

BM helped with the study design, performed analysis, and drafted the manuscript. QL developed the AI tool, contributed data analysis, and writing. MB, IG, PM, JP, KE, and JW contributed to data collection, interpretation, and writing. MS, RB, AN, AH, TU, and OR contributed to data collection and writing. TS, KS, PS, EK, ES, and AB contributed to the study design, data collection, interpretation, and writing. All authors contributed to the article and approved the submitted version.

## Supplementary material

Supplementary Figure 1 Summary of VIOLA-AI estimates aggregated by site. The total estimated haematoma volume [mL **(A)**] and number of haematoma clusters [number **(B)**] are provided. Site names are shown on x-axis. S, ICH stroke; T, head injury/TBI.

Supplementary Table 1 Imaging details show the range of non-contrast CT data that were used, which is representative of different clinical imaging protocols.
