# Supplementary figures and images for: Radiological features of brain hemorrhage through automated segmentation from computed tomography in stroke and traumatic brain injury
Source: Front Neurol. 2023 Sep 28;14:1244672. doi: 10.3389/fneur.2023.1244672 (PMC10568013; doi:10.3389/fneur.2023.1244672)

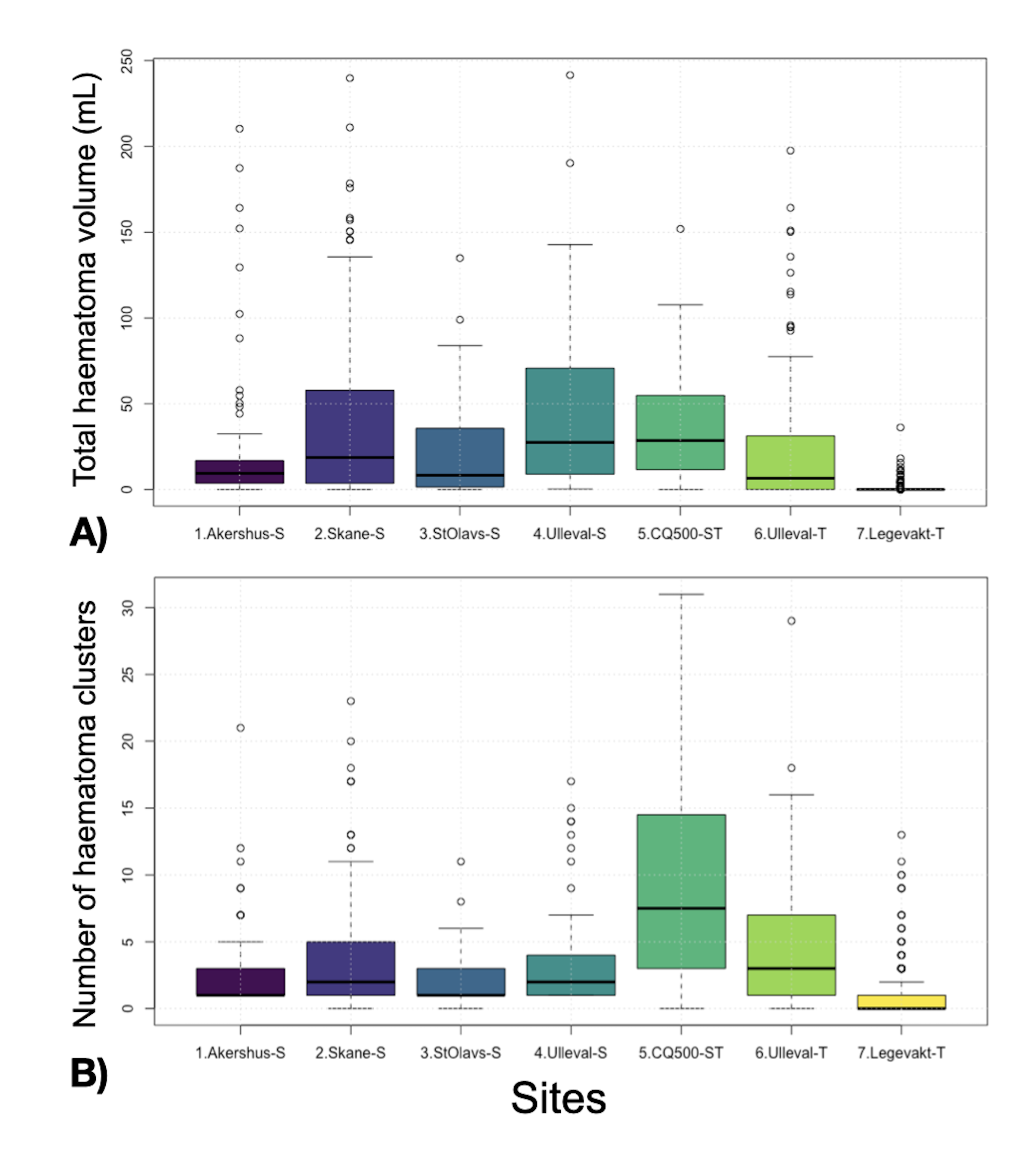

Supplement: Supplementary Figure 1 — Summary of VIOLA-AI estimates aggregated by site. The total estimated haematoma volume [mL (A)] and number of haematoma clusters [number (B)] are provided. Site names are shown on x-axis. S, ICH stroke; T, head injury/TBI. [file Image_1.TIFF]
