# Supplementary material for: Radiological features of brain hemorrhage through automated segmentation from computed tomography in stroke and traumatic brain injury
Source: Front Neurol. 2023 Sep 28;14:1244672. doi: 10.3389/fneur.2023.1244672 (PMC10568013; doi:10.3389/fneur.2023.1244672)
Supplement: Supplementary Table 1 — Imaging details show the range of non-contrast CT data that were used, which is representative of different clinical imaging protocols. [file Table_1.DOCX]

|  | **ICH** | | | | **Mixed** | **TBI** | |
| --- | --- | --- | --- | --- | --- | --- | --- |
| **Group** | **Akershus** | **Skåne** | **NorCoast** | **Ullevål** | **CQ500** | **Ullevål** | **Oslo Emergency** |
| Spatial resolution (median) | 0.43 x 0.43 x 0.50 | 0.43 x 0.43 x 0.60 | 0.42 x 0.42 x  3.0 | 0.38 x 0.38 x  3.0 | 0.49 x 0.49 x 0.62 | 0.38 x 0.38 x  3.0 | 0.42 x 0.42 x  3.0 |
| Spatial resolution (range) | 0.37 to 0.51;  0.37 to 0.51;  0.44 to 1.00 | 0.29 to 0.54; 0.29 to 0.54; 0.29 to 4.00 | 0.30 to 0.53; 0.30 to 0.53; 0.60 to 5.00 | 0.30 to 0.52; 0.30 to 0.52; 0.30 to 5.00 | 0.42 to 0.79; 0.42 to 0.79; 0.62 to 5.00 | 0.29 to 0.53; 0.29 to 0.53; 0.70 to 4.00 | 0.25 to 0.57; 0.25 to 0.57; 2.17 to 6.00 |
| Number of slices (median; range) | 296;  144 to 399 | 296;  45 to 681 | 53;  31 to 260 | 55;  29 to 541 | 256;  51 to 288 | 57;  36 to 259 | 53;  31 to 91 |

**Supplementary table:** Imaging details show the range of non-contrast CT data that were used, which is representative of different clinical imaging protocols.
